# Demographic Variation of *Wolbachia* Infection in the Endangered Mitchell’s Satyr Butterfly

**DOI:** 10.3390/insects8020050

**Published:** 2017-05-09

**Authors:** Jennifer Fenner, Jennifer Seltzer, Scott Peyton, Heather Sullivan, Peter Tolson, Ryan P. Walsh, JoVonn Hill, Brian A. Counterman

**Affiliations:** 1 Department of Biological Sciences, Mississippi State University, Starkville, MS 39762, USA; jls1393@msstate.edu; 2 Department of Biochemistry, Molecular Biology, Entomology and Plant Pathology, Mississippi State University, Starkville, MS 39762, USA; jls30@entomology.msstate.edu (J.S.); jgh4@entomology.msstate.edu (J.H.); 3 Mississippi Natural Heritage Program, Mississippi Department of Wildlife Fisheries and Parks, Jackson, MS 39202, USA; Scott.Peyton@MMNS.state.MS.US (S.P.); Heather.Sullivan@MMNS.state.MS.US (H.S.); 4 The Toledo Zoo, Toledo, OH 43614, USA; peter.tolson@toledozoo.org (P.T.); Ryan.Walsh@toledozoo.org (R.P.W.)

**Keywords:** endosymbiont, cytoplasmic incompatibility, captive rearing, conservation, nymphalid

## Abstract

The Mitchell’s satyr, *Neonympha mitchellii*, is an endangered species that is limited to highly isolated habitats in the northern and southern United States. Conservation strategies for isolated endangered species often implement captive breeding and translocation programs for repopulation. However, these programs risk increasing the spread of harmful pathogens, such as the bacterial endosymbiont *Wolbachia*. *Wolbachia* can manipulate the host’s reproduction leading to incompatibilities between infected and uninfected hosts. This study uses molecular methods to screen for *Wolbachia* presence across the distribution of the Mitchell’s satyr and its subspecies, St. Francis satyr, which are both federally listed as endangered and are considered two of the rarest butterflies in North America. The screens confirmed the presence of *Wolbachia* in the northern and newly discovered southern populations of the Mitchell’s satyr, but not in the St. Francis satyr population. These results combined with previous reports of *Wolbachia* in *N. mitchellii,* highlight that *Wolbachia* infection varies both geographically and temporally in satyr populations. The temporal variance shows the importance of continued monitoring of *Wolbachia* infection during conservation programs. To reduce the risk of reproductive incompatibilities, it is advised that all individuals collected for conservation purposes be screened for *Wolbachia* and recommended to avoid the use of infected individuals for captive breeding and translocation programs.

## 1. Introduction

The Mitchell’s satyr, *Neonympha mitchellii mitchellii* (French), is often cited as one of the rarest butterflies in North America [1,2,3] and was first described from southern Michigan [4]. Until recently, the known distribution of *N. m. mitchellii* included Indiana and Michigan, with historic, now extinct, populations in Ohio, New Jersey, and Maryland. The St. Francis Satyr, *N. mitchellii francisci*, is restricted to a single location in Fort Bragg, North Carolina and differs from other *N. mitchellii* populations in several traits, including male genitalia, wing coloration and habitat [1]. Due to the limited number of extant populations of *N. m. mitchellii* (17 in Michigan and two in Indiana), its extirpation in three states, and the restriction of *N. m. francisci* to Fort Bragg, both subspecies were placed on the endangered species list and given federal protection [5].

**Figure 1:**
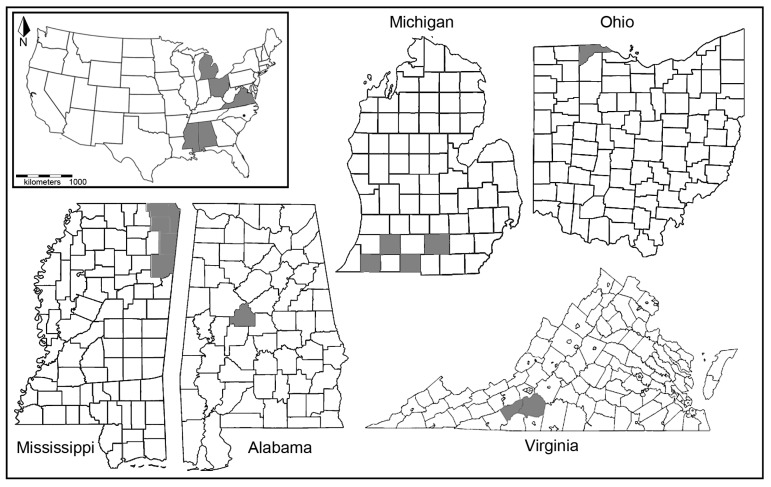
State and county distributions of *N. mitchellii*. Insert of Unites States of America shows states with *N. m. mitchelli* present in grey, and a black dot for *N. m. francisci* presence. Grey regions reflect counties *N. m. mitchelli* were sampled for this study. Black filled circle in North Carolina denotes Fort Bragg that *N. m. francisci* is present.

In 2000 and 2001, eight populations of *N. m. mitchelli* were documented in east-central Alabama [6]. In 2003, a curator of the Mississippi Entomological Museum (MEM), Terence Schiefer, discovered three populations in northeast Mississippi along the Natchez Trace Parkway. In 2010, staff from the Mississippi Museum of Natural History and MEM initiated surveys to determine the distribution of *N. m. mitchellii* in Mississippi. From 2010 to 2014, surveys of small, open to partially wooded wetlands located Mitchell’s Satyrs at 15 sites across Tishomingo, Itawamba, eastern Prentiss, eastern Alcorn, and Monroe counties in Mississippi (Figure 1). The United States Fish and Wildlife Service now includes these southern populations under the Endangered Species Act.

Both subspecies of this butterfly are associated with the sedge-dominated edges of wetlands, where an open canopy is present. Several species of sedges (*Carex* spp.) have been recorded as larval host plants [7,8,9,10]. Northern populations of *N. m. mitchellii* inhabit prairie fens, a relatively stable habitat, whereas the southern populations are generally associated with ephemeral habitats such as open to partially wooded, small wetlands near streams, along the wooded borders, or on the edges of wetlands associated with beaver activity [6,9]. In North Carolina, the constant interruption of plant community succession by ordnance use, prescribed fire, and beaver impoundments has resulted in the persistence of suitable habitat for *N*. *m*. *francisci* at Fort Bragg [10]. Northern populations of *N. m. mitchellii* are univoltine with just one generation per year, whereas southern populations and those of *N*. *m*. *francisci* are bivoltine, having two generations per year. In Mississippi, the first flight period is during early to mid-June, followed by a second flight period in late August. The two subspecies also show clear population genetic differences, however the recently discovered southern populations were genetically indistinguishable from *N. m. francisci* at one mitochondrial and five nuclear markers [11]. Based on this, the recommendation has been for both subspecies to continue to be managed as separate endangered species.

Captive breeding and translocation programs have become common management strategies for repopulating endangered species that have highly fragmented distributions [12]. However, these strategies can greatly increase the risk of disease transmission. In *Danaus plexippus*, monarchs, the translocation of mass bred individuals for commercial trade can spread spores of the parasite *Ophryocystis elektroscirrha*, which can have lethal effects [13]. In many insects, the transmission of bacterial endosymbionts can also have major impacts on the host population, which can be a concern for conservation. *Wolbachia* (Rickettsiales: Rickettsiaceae) is a maternally inherited bacterial endosymbiont that has a range of hosts from phyla Arthropoda to Nematoda, with as many as 75% of all insect species harboring *Wolbachia* [14,15]. *Wolbachia* has evolved mechanisms to increase its transmission that has major impacts of their host’s reproduction. As a result, host populations often experience highly distorted sex ratios and cytoplasmic incompatibilities (CI) between infected and uninfected individuals [16]. The introduction of infected captive individuals during restoration efforts can result in CI with individuals uninfected or carrying a different *Wolbachia* strain. The introduced CI will further reduce the number of reproducing host individuals and increase the chance of extirpation [17]. Therefore, it is pertinent that conservation strategies for arthropods involve screens for *Wolbachia* in captive rearing and translocation efforts.

Captive rearing has been a key strategy in the conservation of *N. m. mitchellii* and in 2014 *Wolbachia* was first reported in *N. m. mitchellii* [18,19]. This previous study by Hamm et al. [19] tested for *Wolbachia* in *N. m. mitchellii* from a single county in Michigan, and ~11% of the individuals tested positive. Here, we assay for the presence of *Wolbachia* in 63 *N. m. mitchellii* sampled across its range including the captive breeding population, 4 *N. m. francisci,* and 10 *Megisto cymela* (little wood satyr) whose distribution overlaps with *N. mitchellii*. We use these to determine (1) if the captive rearing population shows evidence of *Wolbachia*; (2) if *Wolbachia* remains present in the Michigan populations; (3) if additional northern populations show presence of *Wolbachia*; (4) if *Wolbachia* is present in the newly discovered populations in Mississippi and Alabama and (5) if *Wolbachia* is also present in *N. m. fransicii*. The results of this study will identify populations susceptible for *Wolbachia* induced CI and directly inform conservation managers on best practices for the collection and release *N. mitchelli* during captive rearing and translocation programs.

## 2. Materials and Methods

### 2.1. Specimen Acquisition

Whole body samples were obtained from 76 satyr butterflies across four populations: (i) 35 *Neonympha mitchellii mitchellii* samples from southern populations in Alabama, Mississippi, and Virginia; (ii) 27 *N. m. mitchellii* samples from northern populations in Ohio and Michigan; (iii) four *Neonympha mitchellii francisci* from Fort Bragg; and (iv) 10 samples of *Megisto cymela.* All samples were collected from wild populations except the 19 Ohio samples, which were obtained from a captive breeding colony at the Toledo Zoo. Five of the samples from the Toledo Zoo were obtained in the form of wing snips or whole wings. Specific locations of collection sites have been omitted from the manuscript for the safety of the endangered butterflies, but a map of counties that were sampled for this study is provided in Figure 1.

### 2.2. Sample Preparation

The 72 whole body genomic DNA (gDNA) samples were isolated using a DNA extraction kit, Qiagen DNeasy. GDNA extractions for the five wing snips were conducted using mechanical homogenization with a sterile pellet mixer in 10 nm Tris HCl, 1 nm Ethylenediaminetetraacetic acid (EDTA), pH = 8 (TE) and left overnight at room temperature [20]. All gDNA samples were stored at −20 °C.

### 2.3. Wolbachia Screens

Two concurrent Polymerase Chain Reaction (PCR) based assays were required to confirm the absence/presence of *Wolbachia* [17]. The first PCR assayed was a control to confirm DNA extraction was successful, by amplifying the arthropod-specific 28s rRNA gene. Primers used for the 28s PCRs were (28sF3633: 5′ TAC CGT GAG GGA AAG TTG AAA 3′; 28sR4076: 5′ AGA CTC CTT GGT CCG TGT TT 3′) [21]. Those samples that did not amplify for 28s were not used in subsequent analyses. To assay for *Wolbachia* presence, we used *Wolbachia* specific primers to amplify the 16s rRNA gene (WSpec), (WSpecF: 5′CAT ACC TAT TCG AAG GGA TAG 3′ and WSpecR: 5′AGC TTC GAG TGA AAC CAA TTC 3′) [15,22]. Standard PCR protocols were followed using 10 μm forward and reverse primers, 10 mM dNTP PCR grade Mix (Invitrogen), and *Taq* DNA polymerase with 10× Standard *Taq* Buffer (New England Biolabs) with the suggested routine thermocycling conditions for *Taq* (New England Biolabs) and a 52 °C annealing temperature. PCR products were visualized alongside a 100 base pair ladder on a 1% agarose gel run at 100 volts for one hour.

### 2.4. Wolbachia Confirmation

Samples that tested positive for PCR amplification of Wspec were cleaned with ExoSAP-IT (Thermo Fisher Scientific, Waltham, MA, USA) to remove excess nucleotides. Forward and reverse cycle sequencing reactions were run separately using BigDye Terminators (Applied Biosystems) and the Wspec primers. Dye terminator removal and sequencing was conducted at Georgia Genomics Institute. Chromatogram files for each sequence were cleaned by removing low quality and aligned using Sequencher version 5.5.1 (Gene Codes, Ann Arbor, MI, USA). Consensus sequences were generated from the forward and reverse sequences for each sample. To confirm the amplified 16s fragments were from *Wolbachia*, the consensus sequences were used to conduct TBLASTX [23] sequence similarity comparisons against the National Center for Biotechnology Information (NCBI) nucleotide database restricted to sequences from (i) bacteria only and (ii) *Wolbachia pipientis* only. The sequence for the 16s fragment isolated from *N. mitchellii* and *M. cymela* is available at NCBI genbank (Accession # MF002138 and MF002139).

## 3. Results and Discussion

### 3.1. Wolbachia Presence

For all 76 samples tested, the 28s arthropod rRNA gene was successfully amplified. Of these 76 samples, only 11 showed amplification of the *Wolbachia* 16s ribosomal RNA gene. Sequence of the 16s PCR product from positive *N. m. mitchellii* showed the greatest similarity to *Wolbachia* strains isolated from insects (*Drosophila simulans* or to *Maculinea teleius*, the Large Scale Blue Butterfly). The 16s sequences alone were not sufficient to distinguish between *Wolbachia* strains found in insects [24].

### 3.2. Wolbachia Demographics in N. mitchellii

*Wolbachia* infection was variable among the northern and southern *N. m. mitchellii* populations (Table 1, Figure 1). Our results re-confirm the presence of *Wolbachia* in Michigan populations [19]. Two of eight individuals sampled in Michigan tested positive for *Wolbachia* presence. One of these individuals was collected from Jackson county, which is the same county Hamm et al. [19] first reported *Wolbachia* presence among ~11% of the tested *N. m. mitchellii.* This suggests that *Wolbachia* infections can remain variable in *N. m. mitchellii* populations without being lost or spread among all individuals. We report the first evidence of *Wolbachia* outside Jackson Co., in Cass Co., Michigan. Unfortunately, only a single individual was available for genetic testing from Cass Co., but four individuals from two other counties in Michigan were all negative for *Wolbachia*. Collectively, these findings suggest there are likely barriers to *Wolbachia* transmission in the Michigan *N. m. mitchellii* populations, which could be the result of incompatibilities between infected and uninfected individuals.

We confirmed that *Wolbachia* is not present in any of the 19 individuals from the Toledo Zoo captive rearing colony. If *Wolbachia* were present in the Zoo population at a similar frequency as the Michigan populations (~11%–25%), then at least one or a few individuals would be expected to test positive for *Wolbachia*. The lack of *Wolbachia* presence in the 19 individuals strongly suggests *Wolbachia* is either absent or present at negligible levels in the Toledo Zoo captive rearing colony.

Southern populations of *N. m. mitchelli* samples tested positive for the presence of *Wolbachia*. However, at no single southern location (e.g., county) were all *N.m. mitchellii* individuals positive for *Wolbachia*. Five of the 20 individuals collected from Mississippi tested positive for *Wolbachia* (Table 1). Four of the five positive samples were collected from the same area in Monroe County, on the same day. The additional positive individual was collected in Prentis Co., over 300 km from the collections in Monroe Co. Three other individuals collected in Prentis were all negative for *Wolbachia*. Eight additional individuals collected across four different counties also tested negative for *Wolbachia*. Again, these results suggest there may be limited transmission of *Wolbachia* within and between local *N. m. mitchellii* populations.

In Alabama and Virginia populations, no *N. m. mitchellii* were positive for *Wolbachia*. In Alabama, individuals included in this study were only sampled from a single county, however all 11 tested individuals were negative for *Wolbachia* (Table 1). In Virginia, four individuals collected from two counties were all negative. These results suggest that *Wolbachia* infection varies within both northern and southern *N. m. mitchellii* populations. Including more *N. m. mitchellii* individuals from each population and sampling additional loci from *Wolbachia* is needed to accurately estimate the frequency of *Wolbachia* infection and identify potential strain incompatibilities. However, *Wolbachia* was repeatedly found in southern populations of the little brown satyr, *M. cymela*, despite only sampling 1–3 individuals per county. In Mississippi, *Wolbachia* was only present in a portion of the *M. cymela* individuals (Table 1), which was very similar to *Wolbachia* presence in Mississippi populations of *N. m. mitchellii*. Unlike *N. m. mitchellii, M. cymela* in Alabama and Virginia tested positive for *Wolbachia.* This confirms that *Wolbachia* is present in these regions; however, there appears to be limited transmission between these satyr species. *Wolbachia* presence was also confirmed in a Texas population of *M. cymela*, demonstrating the broad range of *Wolbachia* presence among the satyrs.

None of the *N. m. francisci* samples were positive in this study. These results are in stark contract with the study by Hamm et al. [19], which found that all four *N.m. francisci* tested were positive for *Wolbachia.* Instead, here we found that the four individuals we sampled were negative for *Wolbachia*. The lack of *Wolbachia* found in our samples suggests that *Wolbachia* presence may vary temporally and no longer be present in *N. m. francisci.* Alternatively, if the two studies are considered collectively, regardless of when collected, then 50% of the St. Francis would be estimated to be infected with *Wolbachia*. These alternative explanations are difficult to discern with the small sample sizes of St. Francis samples in each study, however there is only a 6.25% chance of sampling four positive (or four negative) individuals from a population with 50% infection. Regardless, our results confirm that *Wolbachia* infection is not ubiquitous throughout the range of *N. m. francisci*.

## 4. Conclusions

The confirmation of *Wolbachia* presence in northern and southern populations of *N. m. mitchellii* could pose major concern for captive breeding and translocation programs. Genetic differences between *N. m. mitchellii* populations and population size estimates suggest that there has been little historical migration between the northern and southern populations due to habitat loss and fragmentation [7,8,11]. If *Wolbachia* transmission also decreased with the Mitchell satyr populations, there is a risk of the evolution of incompatibilities between strains infecting northern versus southern populations. Therefore, we recommend that live *N. m. mitchellii* individuals that show the presence of *Wolbachia* not be translocated between northern and southern populations. To reduce the possibility of CI, we also recommend avoiding any populations with *Wolbachia* positive individuals for captive breeding and translocation programs. The difference in *Wolbachia* presence between the present study and Hamm et al. [19] suggests that *Wolbachia* can vary temporally and that all collected samples for conservation purposes should be screened, regardless of previous *Wolbachia* presence at the collection locations. The use of wing snip sampling can allow for more individuals within a population to be tested without sacrificing individuals, but due to the small sample size of wing snips and low numbers of infection in this study, further investigations should be conducted to discern the method’s effectiveness in other species of concern. We urge researchers and conservation managers to use caution during survey and collection efforts, in order to minimize the potential spread and introduction of *Wolbachia*. Conservation efforts for the Mitchell’s satyr and St. Francis satyr would benefit from further studies of potential CI between infected and uninfected individuals and a detailed examination of *Wolbachia* strain variation among the populations and subspecies.

## Figures and Tables

**Table 1:** *Wolbachia* presence in *N. mitchelli* populations.

Taxa	State	County	Number Tested	Number Positive for *Wolbachia*
*Neonympha mitchelli mitchelli*	AL	Bibb	11	-
	MS	Alcorn	1	-
	MS	Prentis	4	1
	MS	Tishomingo	6	-
	MS	Itawamba	1	-
	MS	Monroe	8	4
	VA	Floyd	2	-
	VA	Franklin	2	-
	MI	Branch	2	-
	MI	Cass	1	1
	MI	Jackson	3	1
	MI	Kalamazoo	2	-
	OH	Toledo Zoo	19	-
*Neonympha mitchelli francisci*	NC	Fort Bragg	4	-
*Megisto cymela*	AL	Baldwin	2	1
	MS	Harrison	2	1
	MS	Tishomingo	3	1
	MS	Wilkininson	1	-
	TX	Blanco	1	1
	VA	Franklin	1	-
